# Optimization of Conditions for Cyanidin-3-*O*-Glucoside (C3G) Nanoliposome Production by Response Surface Methodology and Cellular Uptake Studies in Caco-2 Cells

**DOI:** 10.3390/molecules22030457

**Published:** 2017-03-13

**Authors:** Tisong Liang, Rongfa Guan, Haitao Shen, Qile Xia, Mingqi Liu

**Affiliations:** 1 Zhejiang Proceincial Key Laboratory of Biometrology and Inspection and Quarantine, China Jiliang University, Hangzhou 310018, China; liangtisong@163.com (T.L.); mqliu524@163.com (M.L.); 2 Zhejiang Provincial Center for Disease Control and Prevention, 3399 Binsheng Road, Hangzhou 310051, China; htshen@cdc.zj.cn; 3 Food Science Institute, Zhejiang Academy of Agricultural Sciences, 298 Desheng Road, Hangzhou 310021, China; cookxql@163.com

**Keywords:** C3G, nanoliposomes, response surface methodology, stability, cell viability

## Abstract

We aimed to optimize the formulation of C3G nanoliposomes using response surface methodology. Additionally, we evaluated the stability, particle change, and encapsulation efficiency (EE) of C3G nanoliposomes under different temperatures and storage durations, as well as in simulated gastrointestinal juice (SGF) and simulated intestinal fluid. The morphology of C3G nanoliposomes was observed by transmission electron microscope. The ability of C3G nanoliposomes to affect cancer cell morphology and inhibit cancer cell proliferation was studied with Caco-2 cells. Reverse-phase evaporation method is a simple and efficient method for liposome preparation. The optimal preparation conditions for this method were as follows: C3G concentration of 0.17 mg/mL, phosphatidylcholine/cholesterol ratio of 2.87, and rotary evaporation temperature of 41.41 °C. At optimal conditions, the particle size and EE of the C3G nanoliposomes were 165.78 ± 4.3 nm and 70.43% ± 1.95%, respectively. The C3G nanoliposomes showed an acceptable stability in SGF at 37 °C for 4 h, but were unstable under extended storage durations and high temperatures. Moreover, our results showed that different concentrations of C3G nanoliposomes affected the morphology and inhibited the proliferation of Caco-2 cells.

## 1. Introduction

Cyanidin-3-glucoside (C3G) is a major anthocyanin, which is a class of polyphenols abundant in the pigments of numerous colorful fruits and vegetables [1,2,3]. Studies on the use of anthocyanins as valuable natural alternatives to synthetic food colorings have increased in recent years. Moreover, there is evidence that anthocyanins have potential free radical-scavenging activities that prevent low-density lipoprotein oxidation and positively affect cardiovascular diseases, obesity, and inflammation [4,5,6,7,8]. Other studies indicate that C3G can protect against the adverse effects of UVB radiation, inhibit the proliferation and induce the apoptosis of cancer cells, and reduce oxidative stress [9,10,11,12].

The majority of these studies focus on the properties of cyanidin-3-glucoside (C3G). However, there are few studies on C3G nanoliposomes. Nanoliposomes are vesicles in which a small volume of aqueous solution is surrounded by a bilayer phospholipid membrane, which can be used as liposomes and nanoparticles [13,14,15,16]. There have been numerous research studies on the application of liposomes as a protective membrane in food and pharmacological systems. In addition, nanoliposomes have the advantages of nanoparticles, which can improve targeting and absorption by intestinal epithelial cells. Therefore, nanoliposomes can be used as potential carriers in a food system [17,18,19,20,21,22,23].

There are many studies reported the application of nanoliposomes in many fields, but few studies reported the application of C3G nanoliposomes. In our study, we used the nanoliposomes to improve the effectiveness and stabilities of C3G.We studied the properties and stabilities of C3G nanoliposomes. In this study, we provided a new way and theoretical basis for the future study of C3G.

Response surface methodology (RSM) is a combination of mathematical and statistical techniques that is useful for modeling and analysis. RSM analyzes response surface contours to identify optimal process parameters and uses multiple quadratic regression equations to determine the fit between factors and response functions [24,25]. Given its many advantages, RSM has been widely used to optimize and model liposome preparation conditions. Jangde used RSM to investigate the effects of rotary evaporator speed and water bath temperature on the preparation of quercetin-loaded liposomes. Naeem used RSM to investigate the effects of phospholipid formulation on liposomes. In addition, Lu used RSM to study the effects of tea polyphenol/lecithin ratio and phosphate-buffered saline (PBS) pH on the preparation of tea polyphenol nanoliposomes [26,27,28].

This study utilized RSM to determine the optimal conditions for the preparation of C3G nanoliposomes. The effects of the following factors on the size and encapsulation efficiency (EE) of C3G nanoliposomes were evaluated: phosphatidylcholine/cholesterol ratio (PC/CH, *w*/*w*), C3G concentration (*w*/*v*), and temperature (°C). Furthermore, the stability of nanoliposomes was tested in simulated gastric fluid (SGF) and simulated intestinal fluid (SIF), as well as under high temperature. The ability of nanoliposomes to inhibit cell proliferation was studied with Caco-2 cells.

## 2. Results

### 2.1. Fitting the Model

A total of 20 runs were used for optimizing the three individual parameters in the BBD, the experimental and the EE and size of C3G nanoliposomes according to the factorial design. The results were shown in Table 1, which shows the design and the experimental and predicted values. For the corresponding fitting of the explanatory models, the variations of encapsulation efficiency and size were analyzed. The EE and size obtained in terms of coded factors is calculated as follows:

EE (%) = 70.16 + 0.55X_1_ − 0.30X_2_ + 0.83X_3_ − 0.089X_1_X_2_ − 0.019X_1_X_3_ + 0.056X_2_X_3_ − 1.01X_1_^2^ − 0.61X_2_^2^ − 1.17X_3_^2^ (1)

Size = 171.24 + 0.31X_1_ − 0.053X_2_ + 0.0.026X_3_ − 0.083X_1_X_2_ + 0.28X_1_X_3_ + 0.17X_2_X_3_ − 1.39X_1_^2^ − 0.80X_2_^2^ − 1.55X_3_^2^ (2)

The statistical significance of the models was evaluated by regression analysis and the analysis of variance (ANOVA). The estimated regression coefficients for the response variable, along with the corresponding R^2^, F value, adjusted R^2^ (adj-R^2^), and p value of lack of fit, these results are shown in Table 2.

From Table 2, The Model F-values of 70.16 and 172.14, implied the model was significant. There was only a 0.01% and 0.045% chance that a “Model F-Value” this large could occur due to noise. Values of “Prob. > F” less than 0.05 indicated model terms were significant [29], the independent variable and the linear relationship are significant. The lack of fit of every model was checked used the determination coefficient. Also, the lack of fit showed that the models failed to represent the data in the experimental domain at which points were not included in the regression [30]. The lack of fit of the EE and size were 0.56 and 0.46, which were not significant (*p* > 0.05) for the response surface model, indicating that the model represented the data accurately.

Closer to unity R^2^ value indicated better empirical model fit to actual data. The R^2^ values for the response variable of the EE and size were 0.96 and 0.85, which were higher than 0.80, indicating that the regression models were suitable to explain the behavior, but a large R^2^ value does not always imply the adequacy of the model. Adding a variable to the model will always increase R^2^, regardless of whether the additional variable is statistically significant or not. Thus, it is better to use an adj-R^2^ to evaluate the model adequacy [30]. In Table 2, the R^2^ and adj-R^2^ values of the model are 0.9587, 0.8465, 0.9216 and 0.7883 , indicating that the model fits well with the experimental data and the model can be used to analysis and predict the results of generate C3G nanoliposomes.

### 2.2. Particle Size and PDI

The significance of every coefficient was determined on the basis of *p* values. A lower *p* value indicates that the corresponding coefficient is significant; thus, *p* values less than 0.05 indicate that the model terms are significant.

The results shown in Table 2 are based on the sum of squares. The influence of the independent variables on yield was ranked in the following order: PC/CH ratio > C3G concentration > rotary evaporation temperature.

**Figure 1:**
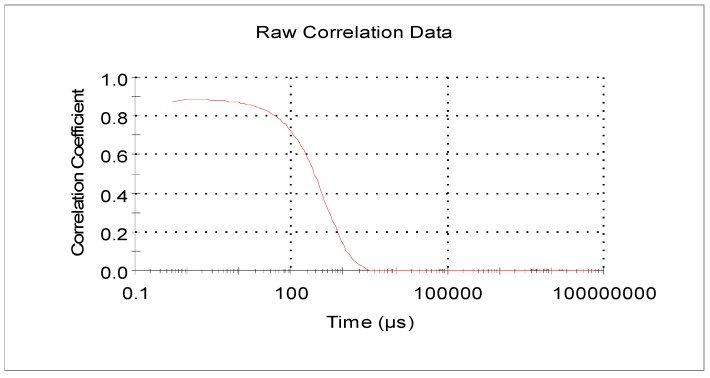
Correlation of the scattered light intensity in nanoliposome solution as a function of time.

Figure 1 presents the correlation between scattered light intensity in the nanoliposome solution as a function of time, as well as the correlation of the scattered light signal intensity at time = t and at different time points. The following points were inferred from the correlogram: first, the correlation between light intensity and time decreased with time and eventually approached zero because of the random Brownian movement of the liposomal particles. Second, the smooth baseline at the end indicated that there was no sedimentation in the sample. Last, the correlogram also showed that the particles had relatively large sizes because the signal changed slowly and the correlation persisted for a longer time (levelling off period) before decaying [31].

**Figure 2:**
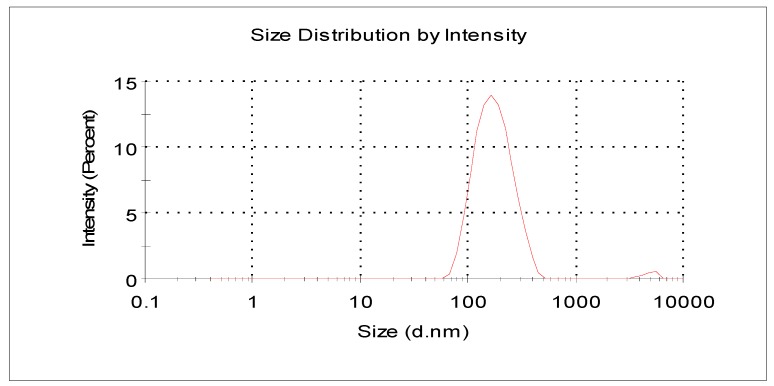
The particle size of the optimized C3G nanoliposomes.

Figure 2 shows that the size distribution curve of the C3G liposomes was a normalized curve that revealed the size variation among particles. Particle sizes ranged from 100 nm to 1000 nm. Particle size is dependent on preparation conditions and materials and can be reduced by energy input in the form of sonic energy (sonication) and mechanical energy (extrusion) [31]. Our study found that the mean particle size of C3G nanoliposomes was 165.78 ± 4.3 nm and the mean PDI of the prepared C3G nanoliposomes was 0.143 ± 0.025.

**Figure 3:**
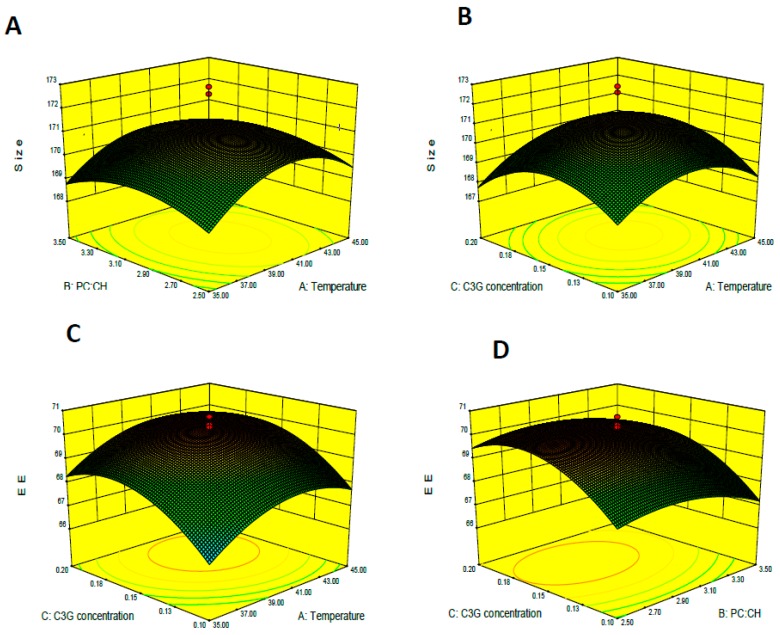
Response surface for the effects of independent variables on encapsulation efficiency and size of C3G nanoliposomes. The effects of phosphatidylcholine-to-cholesterol ratio and temperature on size are shown in (**A**) (C3G concentration = 0.15 mg/mL); The effects of C3G concentration and rotary evaporation temperature were shown in (**B**) (phosphatidylcholine-to-cholesterol ratio = 3); The effects of phosphatidylcholine-to-cholesterol ratio and temperature on EE (%) are shown in (**C**) (phosphatidylcholine-to-cholesterol ratio = 3); The effects of phosphatidylcholine-to-cholesterol ratio and C3G concentration were shown in (**D**) (rotary evaporation temperature = 40 °C).

Figure 3A shows size variation with PC/CH ratio and C3G concentration. The particle size of the C3G nanoliposomes decreased as PC concentration decreased because phospholipids constituted the liposome membrane and PC concentration directly affected the particle size of the liposome. Figure 3B shows the effect of C3G concentration and rotary evaporation temperature on nanoliposome size. Rotary evaporation temperature affected liposome size. Zhou et al. reported that the temperatures of lipid solutions are critical parameters for the properties of gemcitabine liposome injection during preparation [32]. Meanwhile, CO_2_ solubility decreased as temperature increased. Therefore, the dispersion effect of CO_2_ weakened because less CO_2_ was solubilized within the bilayers, which resulted in the formation of larger particles [33].

### 2.3. Encapsulation Efficiency 

Table 2 shows that the linear effects of PC/CH ratio, C3G concentration, and rotary evaporation temperature (°C) were significant (*p* < 0.05). The effects of the independent variables on C3G nanoliposomes are shown in Figure 3. Figure 3C shows that EE increased as PC/CH ratio increased because CH altered the order of lecithin mobility in the lipid bilayer, which reinforces membrane stability [34]. By contrast, EE increased with increased C3G concentration because more C3G was encapsulated into the vesicles (Figure 3D) [35].

These results indicated that higher PC/CH ratios and C3G concentrations increased the EE. In this work, we found that the EE of C3G nanoliposomes was 70.43% ± 1.95%, which is higher than the EE reported in our previous work on nanoliposome preparation [36]. Poor encapsulation may result from the interaction between liposomal membranes and peptides. This interaction destroys the bilayer structure, forms pores, and results in content leakage.

### 2.4. Microscopic Assessment of Nanoliposomes

**Figure 4:**
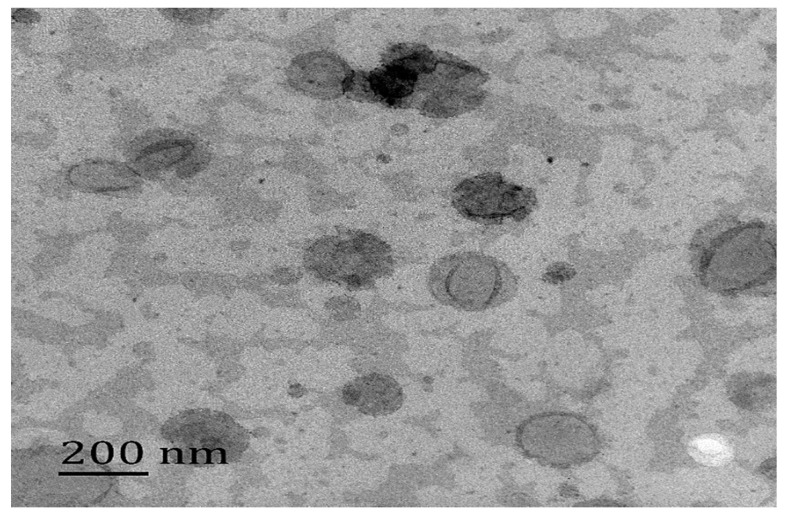
The physical appearance and particle diameter of C3G nanoliosomes.

A TEM study was conducted to investigate the morphology of C3G nanoliposomes. Figure 4 shows a representative recorded TEM image of the C3G nanoliposomes. The nanoparticles exhibited spherical shapes and the size of C3G nanoliposomes is about 200 nm.

### 2.5. In Vitro Release of C3G from Nanoliposomes

**Figure 5:**
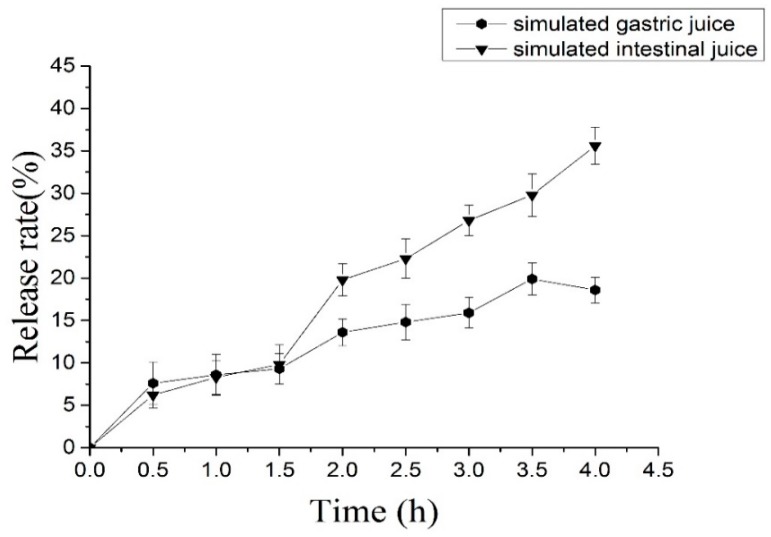
The effect of simulated gastrointestinal juice on C3G nanoliposomes. Data reported are the mean values ± standard variation of three replications.

In vitro release is a crucial surrogate indicator of in vivo performance. C3G nanoliposomes that are used as carriers for the oral administration of C3G must withstand the effect of the digestive system, such as the stomach, intestine, and other organs, given the probable degradation of encapsulated bioactive components by highly acidic conditions and enzymes of SGF. The results presented in Figure 5 shows that approximately 18.6% of C3G was released from nanoliposomes within 4 h in SGF because the nanolipsomes became unstable under proton permeation and low pH. This result indicated the significant protective effects of the lipid bilayer on the core materials [37]. Figure 5 also shows that approximately 35.6% of C3G was released from nanoliposomes within 4 h in SIF. This behavior is characteristic of controlled release. Pancreatic lipase and bile salts in SIF may have released C3G from nanoliposomes. Less C3G was released in SGF than in SIF. These results indicated that the nanoliposomes are acceptably stable and are fit for oral administration [38,39].

### 2.6. Storage Stability Studies

Liposomal product quality is predicted by storage stability, which is a major parameter for liposomal quality. The most promising liposomal system with desirable efficacy is characterized by slow leakage and high stability. Therefore, the evaluation of drug leakage and liposome aggregation or sedimentation during storage is highly crucial to the construction of an optimal liposomal drug delivery system [40,41]. The maintenance of a constant size and high EE over an extended period are indicators of liposome stability as a drug delivery system. Table 3 shows the results of particle size and EE of C3G nanoliposomes under different storage durations.

Table 3 shows the measured average diameter (nm) of liposomes. The liposomes exhibited some significant differences (*p* < 0.05) in the increase of average diameter over three weeks of storage at 4 °C. Particle size ranged from 165.78 ± 4.3 nm to 240.2 ± 5.6 nm, which is equivalent to a 15.1% increase. In addition, EE exhibited little change with storage time. The results showed that the C3G nanoliposomes exhibited aggregation, integration, and acceptable stability during extended storage [42].

### 2.7. Thermostability Test

**Figure 6:**
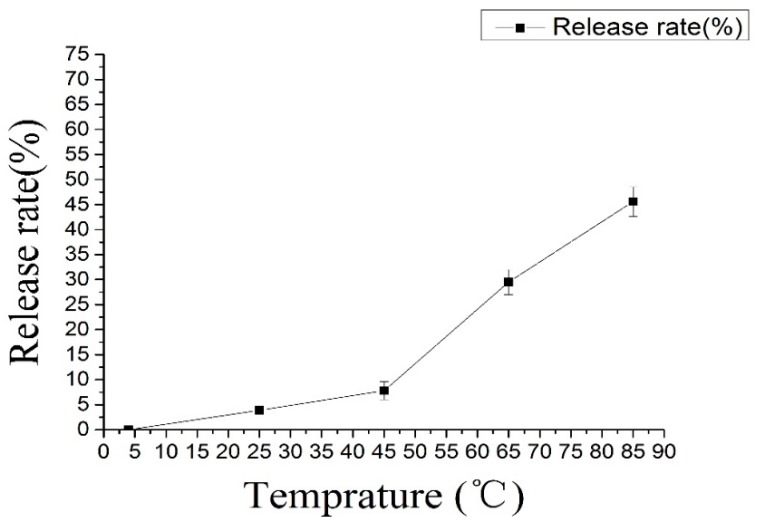
The effect of temprature on nanoliposomes.

The thermal treatment experiments were performed at 4 °C, 25 °C, 45 °C, 65 °C and 85 °C; these temperatures are used in industrial treatments [43]. Figure 6 shows the release rates of C3G nanoliposomes at different temperatures, and that release rate increased as temperature increased. Samples showed the highest loss under 85 °C. At this temperature, more than 65% of C3G nanoliposomes decomposed within 30 min. C3G nanoliposomes were relatively stable at 4 °C to 40 °C. These results indicated that C3G nanoliposomes should be stored at lower temperatures.

### 2.8. Optimization

After investigating the effects of PC/CH ratio, C3G concentration, and temperature on C3G nanoliposomes, the optimum ranges for each independent variable were determined to prepare C3G nanoliposomes with the highest EE. The optimal conditions were as follows: PC/CH ratio of 2.87, C3G concentration of 0.17 mg/mL, and rotary evaporation temperature of 41.41 °C. At these conditions, EE was the highest (70.42%) and the experimental values were closest to the predicted values. Table 4 shows that the deviation was 1.5% and 0.3%, which indicated that the optimized preparation conditions are reliable. C3G nanoliposomes synthesized with the optimized formulation were used in the determination of particle size distribution (Figure 2). Particle size was 165.78 ± 4.3 nm at optimum conditions, which indicated the nanoscale size of the prepared liposomes.

### 2.9. Cell Morphology

**Figure 7:**
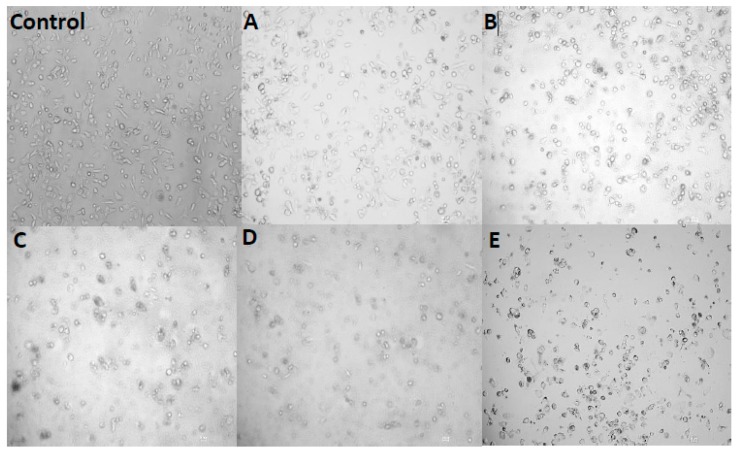
Cell morphology of Caco-2 cells treated with different concentrations of C3G nanoliposomes. The cells were treated with C3G nanoliposomes of different concentration (0 mg/mL (**Control**), 0.05 mg/mL (**A**); 0.10 mg/mL (**B**); 0.15 mg/mL (**C**); 0.20 mg/mL (**D**) and 0.25 mg/mL (**E**)).

Caco-2 cells were incubated with 0, 0.05, 0.10, 0.15, 0.20 and 0.25 mg/mL C3G nanoliposomes for 12 h and then washed with PBS twice. Cell morphology was observed with an inverted microscope at 200× magnification. Figure 7 shows the cell morphology of Caco-2 cells treated with different C3G nanoliposome concentrations. The cells became spherical as the number of cells decreased, which indicated that the C3G nanoliposomes significantly affected the morphology of Caco-2 cells. Compared with the control, cells cultivated with low doses of C3G nanoliposomes (Figure 7A, 0.05 mg/mL) were similar in appearance to control cells and had brownish particles, which were most likely associated with the cell membranes. This result indicated that cells were unaffected by lower doses of C3G nanoliposomes. The cells began to shrink and became irregularly shaped as C3G nanoliposome concentration increased. When C3G nanoliposome concentrations reached 0.20 mg/ml (Figure 7D), the number of cells decreased and apoptosis and necrosis were observed. Microscopic studies indicated that cells exposed to C3G nanoliposomes at higher doses became abnormal in size, acquired an irregular shape, and displayed cellular shrinkage. These results indicated that C3G nanoliposomes can inhibit the proliferation and induce the apoptosis of cancer cells.

### 2.10. Cell Viability

**Figure 8:**
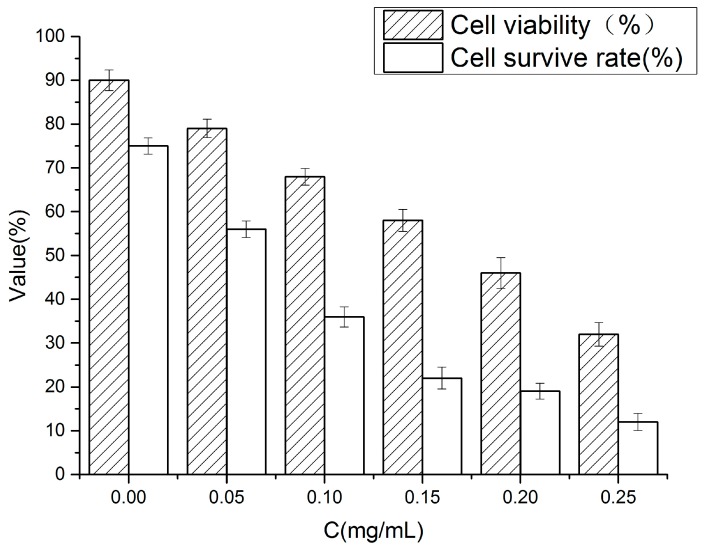
Cell survival rate and cell viability of Caco-2 cells treated with different concenteations of C3G nanoliposomes.

Cell viability was evaluated by the Water Soluble Tetrazolium Salt (WST-1) method. Figure 8 shows the variation in the cellular activity of Caco-2 cells that were exposed to 0, 0.05, 0.10, 0.15, 0.20 and 0.25 mg/mL of C3G nanoliposomes for 12 h.

In Figure 8 cells incubated without nanoliposomes were the blank control. C3G nanoliposome concentrations of 0 to 0.25 mg/mL significantly (*p* < 0.01) inhibited the proliferation of Caco-2 cells. Cell viability became dose-dependent (Figure 8) when cells were exposed to C3G nanoliposome concentrations of 0.05 mg/mL to 0.25 g/mL for 12 h. Percentage (%) WST-1 relative to the control after 12 h of exposure to C3G nanoliposome concentrations of 0.5, 0.10, 0.15, 0.20 and 0.25 mg/mL were 90.11%, 79.21%, 68.32%, 57.93%, 46.15% and 32.56%, respectively. The IC_50_ value of Caco-2 cells exposed to C3G nanoliposomes was 0.19 ± 0.02 mg/mL.

### 2.11. Cell Survival Rate

Viability shows cell mitochondrial activity, whereas survival rate shows the percentage of viable cells. In our work, cell survival rate was evaluated by trypan blue exclusion assay. In this assay, viable cells remain unchanged, whereas dead cells are stained blue. The survival rate was expressed by the percentage of living cells. Figure 8 shows the survival rate of Caco-2 cells treated with different C3G nanoliposome concentrations. Compared with the control, the survival rate decreased as C3G nanoliposome concentration increased. The number of living cells decreased upon exposure to C3G nanoliposomes. Cell survival rate decreased by approximately 60% compared with the control. The results indicated that different concentrations of C3G nanoliposomes decreased the cell survival rate.

## 3. Discussion

We used RSM to optimize the formulation of C3G nanoliposomes. Moreover, we evaluated the stability of C3G nanoliposomes. Particle size and EE% were found to be 165.78 ± 4.3 nm and 70.43% ± 1.95%, which indicated that the nanoliposomes were on a nanoscale level with a high EE. Results of the stability study indicated that the nanolipsosomes were unstable under high temperatures and extended storage. The simple and convenient reverse-phase evaporation method conferred high EE to nanoliposomes. However, the particle size was too high. In the future studies, we will use the C3G nanoliposomes to culture cell to study the effect of nanoliposomes to cells in molecular level, so a smaller size of C3G nanoliposomes is expected, and because the liposomes were used as carriers, the size is smaller, and they are more effective. At the same time, the uniform particle size can improve the stability of liposomes. Therefore, identifying a method that synthesizes nanoliposomes with smaller, uniform particle sizes is necessary. Moreover, a high EE is necessary for nanoliposomes when used as carriers. In this study, the nanoliposomes were unstable under high temperatures and extended storage. Therefore, improving the stability of C3G nanoliposomes is crucial and requires further study.

C3G is a natural pigment that can inhibit UVB-induced oxidative damage and inflammation [9], and is a potent antioxidant that displays anti-cancer properties in vitro and in vivo [10,44,45]. However, few studies have evaluated the antioxidant and anti-cancer properties of C3G nanoliposomes. The results of this study revealed that C3G nanoliposomes inhibited cell proliferation and affected cell morphology. Few studies have evaluated the properties of C3G nanoliposomes, on a molecular level. Therefore, further studies will use a proper cell model to investigate the properties, effects, and mechanisms of C3G nanoliposomes on cancer cells.

## 4. Materials and Methods

### 4.1. Materials

The Caco-2 cells were obtained from CBCAS (Shanghai, China), Cyanidin-3-glucoside (C3G) was purchased from Chengdu Biopurify Phytochemicals Ltd. (Chengdu, China). Phosphatidylcholine (PC) and cholesterol (CH) were purchased from Beijing Shuangxuan Microorganism Co. Ltd. (Beijing, China). Chloroform and diethyl ether were obtained from Hangzhou Jiachen Chemical Company (Hangzhou, China). All other chemicals were of reagent grade. The water used for all experiments was deionized water.

### 4.2. Methods

#### 4.2.1. Preparation of G3G Nanoliposomes

C3G nanoliposomes were prepared by the reverse-phase evaporation method [46,47]. A certain amount of phosphatidylcholine (PC) and cholesterol (CH) were dissolved in chloroform-diethyl ether. C3G was dissolved in PBS (0.20 M, pH 7.4). Then, the organic phase was homogenized with the aqueous phase by probe sonication for 10 min. The mixture was transferred to a round-bottomed flask. The organic solvent was evaporated under reduced pressure with a rotary evaporator to form a gel. Then, 30 mL of phosphate-buffered solution was added to the gel, which was then probe-sonicated for an additional 25 min, and then take to particle size and EE analyses.

#### 4.2.2. Encapsulation Efficiency Determination

The encapsulation efficiency (EE) is defined as the ratio of C3G entrapped in liposomes to that in the delivery system [48], which is an important parameter for nanoliposomes when defined as delivery systems. It was calculated to determine the concentration of entrapped C3G in nanoliposomes and unentrapped C3G in the aqueous phase. The C3G nanoliposomes were separated from the aqueous phase using a freezing centrifuge (GL 20A, Sorvall Biofuge Stratos Co., Fisher Scientific, Leicestershire, UK). A 0.5 mL nanoliposome suspension was taken and spun at 10,000 rpm for 30 min at 4 °C. The same suspension was ruptured using a certain volume of ethanol, and the total amount of C3G was determined spectrophotometrically. The percentage of encapsulation efficiency (EE) was calculated according to Equation (3) [49]:

(3) $$\mathrm{EE}\%=\frac{W_{en}}{W_{total}}\times 100$$

where *W_total_* is the amount of free C3G, and *W_en_* is the total amount of C3G present in 0.5 mL of nanoliposomes (*W_total_* and *W_en_* were measured by spectrophotometer and then calculated).

#### 4.2.3. Particle Size and Polydisperity Index (PDI) Measurement

Nanoliposome particle size and PDI were determined by particle size analyzer (Zetasizer Nano ZS 90, Malvern Company, Malvern, UK). All measurements were performed at 25 °C with non-invasive back-scattering and dynamic light scattering technology. The measurement was repeated thrice per sample for three samples.

#### 4.2.4. Microscopic Assessment of Nanoliposomes

A transmission electron microscope (JEM-2100, Japanese electronics Co., LTD, Tokyo, Japan) was employed to determine the microstructure of C3G nanoliposomes with a negative staining method. A drop of this solution was placed on a Formvar-carbon coated copper grid (200 mesh, 3 mm diameter HF 36) for 5 min and then to image [50].

#### 4.2.5. Storage Stability Studies

To evaluate the stability of C3G nanoliposomes, the samples were stored at a 4 °C environment for 21 days. Then, the samples were removed to measure EE and particle size at different time points. Measurements were performed thrice [51].

#### 4.2.6. Thermal Stability Test

We studied the thermal stability of C3G nanoliposomes under different temperatures (4 °C, 25 °C, 45 °C, 65 °C and 85 °C) at pH 7.4. After 30 min, the C3G nanoliposomes were removed and the EE of each sample was determined [43]. The release ratios were calculated with Equation (4):

(4) $$\mathrm{Release}\;\mathrm{ratio}\%=\left(1-\frac{EE_{T}}{EE_{0}}\right)\times 100$$

where *EE_0_* is the EE of the C3G nanoliposomes after incubation at standard temperature (4 °C) and *EE_T_* is the EE of the C3G nanoliposomes after incubation under different temperatures.

#### 4.2.7. Experimental Design for Response Surface Methodology (RSM)

RSM as an effective tool for optimizing the formulation of C3G nanoliposomes. The optimization was designed based on a three-factor Box-Behnken design with a total of 20 experimental runs [30]. Based on the previous single-factor test, the results showed that the rotary evaporation temperature, C3G concentration and PC/CH ratio has effect on particle size and EE of C3G nanoliposomes, Cholesterol can change the order of mobility of lecithin in the lipid bilayer, thus reinforcing the membrane stability. C3G concentration and the rotary evaporation temperature can affect the EE and size of C3G nanoliposomes.so three factors were selected as key factors responsible for the EE and particle size. As follows, rotary evaporation temperature (X_1_), PC/CH ratio (X_2_), C3G concentration (X_3_). The encapsulation efficiency and particle size were the response values. Table 5 shows the levels of three factors.

The response could be related to the selected variables by a second-order polynomial model. In this study, a second-order polynomial (Equation (5)) was used to generate response surfaces:

(5) $${Ŷ}_{i}=\beta_{0}+\sum_{i}\beta_{i}X_{i}+\sum_{i}\beta_{ii}X_{i}^{2}+\sum_{i\neq j}\beta_{ij}X_{i}X_{j}$$

where ${Ŷ}_{i}$ represents the predicted responses, $X_{i}$ and $X_{j}$ are the coded values of independent variables, *β*_0_ is the intercept responses, $X_{i}$ and $X_{j}$ are the coded values of independent variables, *β*_0_ is the intercept coefficient, *β_i_* are the linear coefficients, *β_ij_* are the squared coefficients, and *β_ij_* are the interaction coefficients [52]. Statistical significance of the terms in the regression equations was examined. The significant terms in the model were found by analysis of variance (ANOVA) for each response. The adequacy of the model was checked accounting for R^2^ and adjusted R^2^. The desired goals for each variable and response were chosen. All the independent variables were kept within the range while the responses were either maximized or minimized.

#### 4.2.8. In Vitro Release of C3G from Nanoliposomes

SGF and SIF were prepared according a previous study [30]. The SGF of pH 1.3 include hydrochloric acid (0.10 M), deionized water and pepsin. The pH value of SGF was made an adjustment by hydrochloric acid (0.10 M).The SIF include potassium dihydrogen phosphate (6.8 mg/mL), sodium hydroxide (0.10 M), deionized water and trypsin (10 mg/mL).The pH value of SIF was adjusted to 7.5 using sodium hydroxide (0.10 M).10 mL of C3G nanoliposome suspensions was mixed with 1 mL simulated gastrointestinal juice in a 50 mL beaker. Then the beaker was placed on a magnetic stirrer adjusted to a constant speed of 150 rpm at 37 °C. Aliquots of 0.2 mL were sampled from the beaker at predetermined intervals. The release of C3G from nanoliposomes was evaluated by a release ratio. The release ratio was calculated using Equation (4) [43]:

(6) $$\mathrm{Release}\;\mathrm{ratio}\%=\left(1-\frac{EE_{T}}{EE_{0}}\right)\times 100$$

where *EE*_0_ is the encapsulation efficiency of C3G nanoliposomes before incubation, and *EE_T_* is the encapsulation efficiency of C3G nanoliposomes after incubation for the time.

#### 4.2.9. Cell Culture

The Caco-2 cells were cultured in DMEM medium (Gibco BRL, Gaitherburg, MD, USA), with 10% fetal calf serum (Thermo Scientific Company, Beijing, China), 2.9 μg/mL L-glutamine (Solarbio Life Sciences, Beijing, China). The cells were cultured at 37 °C in a humidified atmosphere of 5% CO_2_ [53,54].

#### 4.2.10. Cell Morphology

For document morphological changes in Caco-2 cells in response to different concentrations of C3G nanoliposomes, the cells (control and C3G nanoliposomes exposed) were washed with PBS after 12 h of incubation. Phase contrast images of the cells exposed to the C3G nanoliposomes were observered using an inverted microscope (Nikon Eclipse Ti, Nikon, Tokyo, Japan) at 100× magnification [55].

#### 4.2.11. Cell Survival Rate

The Trypan Blue assay (Beyotime Biotechnology, Shanghai, China) was used to evaluate the cell survival rate. The Caco-2 cells were plated in the 12-well plates (1 × 10^4^ cells per well) and incubated for 24 h. Then, the cells were treated with a range of concentrations (0, 0.05, 0.10, 0.15, 0.20 and 0.25 mg/mL) of different C3G nanoliposomes for 24 h. Cells cultured in the free medium were taken as the control. After 24 h, the cells were harvested with 200 μL trypsin-EDTA solution (Solarbio Life Sciences). The mixture of the supernatant and detached cells was centrifuged at 2000 rpm for 2.5 min. The cells were resuspension use the suspension solution. Then, 100 μL cell suepension was added with 100 μL Trypan Blue solution. After 5 min staining, cells were counted using automated cell counter (Bio-Rad, Irvine, CA, USA). Cell survival rate (%) is expressed as percentage of the living cell number/the total cell number [56,57].

#### 4.2.12. Cell Viability Assay

Cell viability was evaluated by the Water Soluble Tetrazolium Salt (WST-1) Cell Proliferation and Cytotoxicity Assay kit (Beyotime Biotechnology) in accordance with the manufacturer’s directions. Caco-2 cells were harvested with 0.25% trypsin-EDTA solution and sub-cultured into 96-well plates, then treated with different concentrations of C3G nanoliposomes for 24 h. At specific time points, 10 μL of the reagent was added to each well containing 100 μL of cell suspension (2 × 10^3^ cells) and incubated for an additional 1–2 h. Cells cultured in complete medium were considered as the control, whereas complete medium without cells served as the blank. Each test was performed in triplicate. Absorbance was measured at 450 nm on a microplate reader. Cell viability was expressed as the absorption percentage of the C3G nano-exposed cells compared with the controls [58,59].

#### 4.2.13. Statistical Analysis

Optimization data on C3G nanoliposome preparation were analyzed by the Design-Expert v 8.0.6 software (Stat-Ease, Inc., Minneapolis, MN, USA). All experiments were performed thrice in duplicate per sample. Data were presented as the means ± standard deviations from at least three independent measurements. Statistical analysis was performed using SPSS version 21.0 for Windows (International Business Machines Corporation, Armonk, NY, USA).

## 5. Conclusions

The effects of C3G concentration, PC/CH ratio, and rotary evaporation temperature (°C) on C3G nanoliposome preparation were studied. We predicted EE and particle size with a second-order polynomial model. The results showed that EE and particle size increased as PC/CH ratio and C3G concentration increased. Moreover, rotary evaporation temperature affected particle size.

The optimum preparation conditions were determined through numerical optimization. The optimal conditions were as follows: C3G concentration of 0.17 mg/mL, PC/CH ratio of 2.87, and rotary evaporation temperature of 41.41 °C. Under these conditions, the experimental EE and size of the C3G nanoliposomes were 70.43% ± 1.95% and 165.78 ± 4.3 nm, respectively, which approached the predicted values.

The stabilities of the C3G nanoliposomes were tested in SGF, SIF, and extended storage, as well as at high temperatures. The nanoliposomes were stable in SGF and SIF at 37 °C for 4 h and under lower storage temperatures. Moreover, we studied the ability of C3G nanoliposomes to inhibit the proliferation and affect the cell morphology of Caco-2 cells. Different C3G nanoliposome concentrations significantly affected cell morphology and inhibited cell proliferation.

## Figures and Tables

**Table 1:** Scheme of CCRD with the responses results of three independent factors.

Run	Independent Variable						
	Temperature (°C)	PC/CH (*w*/*w*)	C3G Concentration (*w*/*v*)	EE (%)		Size (nm)	
				**Actual**	**Predicted**	**Actual**	**Predicted**
1	35.00	2.25	0.10	65.32	65.61	166.1	166.65
2	40.00	3.00	0.15	67.23	67.02	166.40	166.98
3	40.00	3.00	0.15	65.36	64.81	165.40	166.24
4	45.00	3.75	0.20	65.38	65.68	164.80	166.07
5	45.00	2.25	0.10	67.36	67.14	165.80	166.65
6	45.00	2.25	0.20	67.84	68.47	166.80	167.08
7	40.00	3.00	0.07	66.38	66.67	165.70	166.24
8	40.00	4.26	0.15	67.68	67.47	166.60	167.17
9	35.00	3.75	0.10	66.23	66.38	167.30	166.78
10	40.00	3.00	0.15	68.50	68.24	168.90	167.84
11	35.00	2.25	0.20	67.28	67.02	166.50	166.29
12	40.00	1.74	0.15	65.35	65.50	167.40	166.03
13	40.00	3.00	0.23	65.32	65.46	168.20	166.82
14	31.59	3.00	0.15	68.50	68.25	167.10	166.90
15	40.00	3.00	0.15	70.29	70.16	170.90	171.24
16	48.41	3.00	0.15	69.12	70.16	172.60	171.24
17	40.00	3.00	0.15	70.43	70.16	170.40	171.24
18	40.00	3.00	0.15	70.32	70.16	172.90	171.24
19	45.00	3.75	0.10	70.71	70.16	169.70	171.24
20	35.00	3.75	0.20	70.75	70.16	170.70	171.24

**Table 2:** ANOVA and regression coefficients of the second-order polynomial model for the response variables (actual values).

Source	DF	EE%			Size(nm)		
		Coefficient	Sum of Squares	*p* Value	Coefficient	Sum of Squares	*p* Value
Model	9	70.16	68.15	<0.0001	172.14	93.94	0.0045
Linear							
X_1_	1	0.55	4.15	0.0037	0.31	1.35	0.3946
X_2_	1	−0.30	2.81	0.0113	−0.053	0.086	0.8266
X_3_	1	0.83	9.38	0.0002	0.026	8.95× ^−3^	0.9436
Quadratic							
X_1_^2^	1	−1.01	14.60	<0.0001	−1.39	27.89	0.0023
X_2_^2^	1	−0.61	27.33	<0.0001	−0.80	46.53	0.0004
X_3_^2^	1	−1.17	19.64	<0.0001	−1.55	34.64	0.0011
Interaction							
X_1_X_2_	1	−0.089	0.14	0.5008	−0.083	0.12	0.7920
X_1_X_3_	1	−0.019	2.812 × ^−3^	0.9239	0.28	0.61	0.5645
X_2_X_3_	1	0.056	0.056	0.6712	0.17	0.50	0.5999
Residual	10		2.93			17.04	
Lack of fit	5		1.37	0.5580		8.96	0.4563
Pure error	5		1.57			1.62	
Total	19		71.08			110.98	
R^2^		0.9587			0.8465		
Adj-R^2^		0.9216			0.7883		
CV		0.80			0.78		

**Table 3:** The average diameter (particle size) of C3G liposome measured over three weeks consecutively.

Days	0	3	6	9	12	15	18	21
Average Diameter (nm)	165.78 ± 4.3 ^a^	166.78 ± 4.1 ^a^	168.53 ± 3.2 ^a,b^	179.6 ± 2.5 ^c^	189.5 ± 5.3 ^d^	191.3 ± 5.1 ^e^	220.32 ± 3.5 ^f^	240.2 ± 5.6 ^g^
EE (%)	70.43 ± 1.95 ^a^	70.26 ± 1.2 ^a^	70.56 ± 1.5 ^a^	69.95 ± 2.5 ^a^	70.32 ± 1.23 ^a^	70.12 ± 2.5 ^a^	70.62 ± 1.6 ^a^	70.23 ± 2.1 ^a^

Note: Values with different letters indicate significant difference (*p* < 0.05).

**Table 4:** Comparing the predicted values and actual values.

Index	Predicted Value	Actual Value	Deviation%
Size (nm)	168.3 ± 3.5	165.78 ± 4.3	1.5
EE (%)	70.16 ± 2.08	70.43 ± 1.95	0.3

Note: bias (%) = (predicted values − experimental values)/predicted values × 100.

**Table 5:** Predicted optimum conditions for the preparation of C3G nanoliposomes.

Independent Variables	Symbols	Code Levels		
		−1	0	1
Phosphatidylcholine/cholesterol	X_1_	2.5	3.0	3.5
C3G concentration (mg/mL)	X_2_	0.1	0.15	0.2
Rotary evaporation temperature (°C)	X_3_	35.0	40.0	45.0

## Abbreviations

The following abbreviations are used in this manuscript:

Caco-2 cells	Human epithelial colorectal adenocarcinoma cells
DMEM	Dulbecco’s modified eagle medium
C3G	Cyanidin-3-glucoside
RSM	Response surface methodology
SGF	Simulated gastrointestinal fluid
SIF	Simulated intestinal fluid
TEM	Transmission electron microscope
EE	Encapsulation efficiency
ANOVA	Analysis of variance
PC	Phosphatidylcholine
CH	Cholesterol
